# Phase I of the Surveillance for Enteric Fever in Asia Project (SEAP): An Overview and Lessons Learned

**DOI:** 10.1093/infdis/jiy522

**Published:** 2018-10-10

**Authors:** Caitlin Barkume, Kashmira Date, Samir K Saha, Farah Naz Qamar, Dipika Sur, Jason R Andrews, Stephen P Luby, M Imran Khan, Alex Freeman, Mohammad Tahir Yousafzai, Denise Garrett

**Affiliations:** 1 Typhoid Programs, Sabin Vaccine Institute, Washington, D. C; 2 Global Immunization Division, Centers for Disease Control and Prevention, Atlanta, Georgia; 3 Infectious Diseases and Geographic Medicine, Stanford University, California; 4 Child Health Research Foundation, Department of Microbiology, Dhaka Shishu (Children) Hospital, Bangladesh; 5 Department of Pediatrics and Child Health, Aga Khan University, Karachi, Pakistan; 6 Translational Health Science and Technology Institute, Faridabad, India

**Keywords:** Typhoid, paratyphoid, enteric fever, Salmonella, South Asia, antimicrobial resistance

## Abstract

**Objective:**

The objective of Phase I of the Surveillance for Enteric Fever in Asia Project (SEAP), a multiphase surveillance study characterizing the burden of disease in South Asia, was to inform data collection for prospective surveillance and to capture clinical aspects of disease.

**Methods:**

Through a retrospective record review conducted at hospitals in Bangladesh, India, Nepal, and Pakistan, we examined laboratory and clinical records to assess the culture positivity rate for *Salmonella* Typhi and *Salmonella* Paratyphi, age and sex distribution, and antimicrobial susceptability in each country.

**Results:**

Of all blood cultures performed in Bangladesh, India, Nepal, and Pakistan, 1.5%, 0.43%, 2%, and 1.49%, respectively, were positive for *S.* Typhi and 0.24%, 0.1%, 0.5%, and 0.67%, respectively, were positive for *S.* Paratyphi. A higher proportion of laboratory-confirmed infections in Bangladesh and Pakistan were aged ≤5 years, while India and Nepal had a higher proportion of participants aged 15–25 years. In all countries, the sex of the majority of participants was male. The majority of isolates in all countries were resistant to fluoroquinolones, with a high proportion also resistant to ampicillin, chloramphenicol, and trimethoprim-sulfamethoxazole.

**Discussion:**

Enteric fever remains endemic in South Asia. Data generated by this study can help inform strategies for implementation and evaluation of prevention and control measures.

Enteric fever, a preventable illness caused by the organisms *Salmonella enterica* subspecies *enterica* serovar Typhi or serovar Paratyphi, is estimated to cause >15 million illnesses and nearly 153000 deaths worldwide annually [1, 2]. These organisms are transmitted through food and water contaminated with fecal matter, and their control ultimately depends on access to potable water, as well as safely managed sanitation and hygienic practices in homes and in food production. Vaccines that protect against typhoid fever have been available for many years, and in March 2018, the World Health Organization (WHO) recommended the use of a newly prequalified typhoid conjugate vaccine (TCV) for typhoid fever prevention in countries of endemicity [3]. Compared with the 2 previously available vaccines, TCV provides longer-lasting protection, requires fewer doses, and is additionally suitable for children aged 6 months to <2 years, allowing delivery through routine childhood immunization programs [3–5]. Furthermore, the TCV was included in the 2019–2020 funding window of Gavi, the Vaccine Alliance [6]. To deliver the TCV to susceptible populations, it requires the support of country governments in many low- and middle-income countries, as well as other stakeholders, such as Gavi, the Vaccine Alliance. However, the absence of credible estimates of the burden of disease has made it difficult to provide policymakers with accurate estimates of the cost and potential impact of targeted interventions for prevention and control of enteric fever, including TCV.

One contributing factor to uncertainty about the burden of disease is the lack of reliable diagnostic testing. The most consistently valid method for laboratory diagnosis is blood culture, estimated to be only 61% sensitive [7] in a recent meta-analysis, and may be dependent on sample volume [8]. In many low- and middle-income countries, blood culture is also largely unavailable, particularly in rural areas. Additionally, the widespread use of antibiotics to treat typhoid empirically may reduce the sensitivity of blood culture and further reduce estimates of disease burden; in Nepal, 35% of participants had taken antibiotics prior to seeking healthcare at the study hospitals [9].

Over the last 2 decades, several surveillance studies have attempted to characterize the burden of enteric fever, including the Diseases of the Most Impoverished Program and the Typhoid Fever Surveillance in Africa Program [10, 11]. While these studies were among the first prospective, population-based studies that estimated the incidence of enteric fever in Asia and sub-Saharan Africa, they have left several knowledge gaps, including the incidence of specific complications and the spectrum of disease and severity. Data on the cost of illness and the geographic spread of strains, including antimicrobial-resistant ones, are also limited. Since these studies, the Bill and Melinda Gates Foundation has invested in several new surveillance studies, including the Severe Typhoid in Africa project and the Surveillance for Enteric Fever in Asia Project (SEAP). SEAP is a multiphase enteric fever surveillance study designed to fill knowledge gaps about the burden of disease in Bangladesh, India, Nepal, and Pakistan. With the ability to characterize the burden of enteric fever in 3 major cities and 1 peri-urban area within the selected countries, results from this project will be able to help inform the use of TCVs and other typhoid prevention and control efforts.

The project has 2 phases: Phase I, a retrospective record review, and Phase II, a prospective study with defined enrollment criteria, including specific case definitions. Phase I had 3 objectives: (1) identify site hospitals in selected countries where enteric fever is endemic, to assess the burden of disease through record review and inform data collection for Phase II surveillance; (2) ensure that the hospital had capacity to participate in Phase II surveillance; and (3) map cases geographically to confirm the feasibility of creating a well-defined catchment area for each hospital in which the incidence of enteric disease can be estimated during Phase II surveillance. This article will present the results of the first objective.

## METHODS

### 

Based on the available data in the literature on the geographic burden of enteric fever, we chose Bangladesh, India, Nepal, and Pakistan as potential country sites.

### Study Site Selection

We conducted a standard assessment of existing hospitals, health clinics, and laboratories in the area to determine possible study sites. Key variables in the health facility assessment were as follows: (1) existing evidence on the prevalence of enteric fever; (2) a population that is representative of the overall demographic and socioeconomic characteristics of the surrounding population; (3) a strong laboratory capacity and inpatient (medical and surgical) and outpatient facilities; (4) a location geographically close to the population served by the study hospital; (5) the availability of population-based sociodemographic indicators from reliable sources (eg, bureau of statistics of the city/district/national level or demographic health surveys) or the ability to obtain a geographically representative sample, which will allow extrapolation of population size and demographic characteristics; and (6) a willingness and commitment to conduct the study on the basis of agreed upon aims and objectives. Based on this assessment, we chose several sites in each country, specified below, for inclusion in the study.

#### Bangladesh

Two sites were selected in Bangladesh: Dhaka Shishu Hospital, a 640-bed tertiary-care hospital, and Shishu Sasthya Foundation Hospital, a 200-bed hospital providing primary care only. Both serve a catchment area of 3.4 million people and are the 2 largest pediatric hospitals in Dhaka (serving patients aged <18 years). Owing to proximity, both hospitals are within the same catchment area, in an urban and peri-urban setting. Both hospitals are also sentinel sites in the WHO-coordinated Invasive Bacterial Vaccine-Preventable Disease surveillance network, which captures information electronically on hospitalized patients with suspected or laboratory-confirmed pneumonia, sepsis, and meningitis. The hospitals were able to use this surveillance network to create a platform to capture enteric fever cases, which facilitated data collection for Phase I.

#### India

Five sites were selected in India: the Postgraduate Institute of Medical Sciences, a mixed public-private tertiary hospital in Chandigarh with 1960 beds; Medanta Hospital, a private tertiary hospital in Gurugram (previously known as Gurgaon), Haryana, with 1250 beds; Christian Medical College, a private tertiary hospital in Vellore, Tamil Nadu, with 2800 beds; Apollo Gleneagles Hospital, a private tertiary hospital in Kolkata, West Bengal, with 750 beds; and Kasturba Medical College – Manipal University Hospital, a private tertiary hospital in Manipal, Karnataka, with around 2000 beds. All sites serve urban populations, except Kasturba Medical College, which serves a peri-urban population.

#### Nepal

Sites were chosen because of a prospective enteric fever surveillance study already in place [9], which facilitated data collection. The 4 selected sites were: Bayalpata Hospital, a 15-bed hospital serving a rural population in Achlam; Damauli Hospital, a district hospital serving the town of Vyas, which has a population of 43000; Kirnetar Health Center, a clinic in rural Dolakha; and Dhulikhel Hospital in Kavrepalanchowk, serving a peri-urban population in the greater Kathmandu Valley and with catchment area of approximately 330000 people.

#### Pakistan

Three sites in Pakistan were selected, consisting of the 3 hospitals in the Aga Khan University Hospital network: Aga Khan University Hospital, a 700-bed tertiary care hospital in Karachi; Aga Khan Hospital for Women and Children, a 48-bed secondary care hospital in Karachi; and Aga Khan Hospital Hyderabad, an 87-bed secondary care hospital in Hyderabad. Both Aga Khan Hospital for Women and Children and Aga Khan Hospital Hyderabad only provide care for women and children. These hospitals cover a catchment area of 3 million people.

### Study Period, Data Sources, and Case Definitions

After site selection was completed, we conducted a retrospective review of data available on enteric fever from the selected hospitals, including suspected cases and blood culture–confirmed *S*. Typhi and *S.* Paratyphi infection. A minimum of 2 years of data were collected to assess any seasonal variation at the selected sites. Specific methods for the sites, including case definitions, study periods, and data sources, are described in Table 1.

**Table 1.** Sentinel Healthcare Facilities, Study Period, Case Definitions, and Data Sources in Bangladesh, India, Nepal, and Pakistan, Surveillance for Enteric Fever in Asia Project (SEAP) Phase I, 2012–2016

Country	Sentinel Healthcare Facilities	Study Period	Case Definitions	Data Sources
Bangladesh	Dhaka Shishu Hospital and Shishu Sasthya Foundation Hospital, Dhaka	January 2013– December 2014	Suspected case: a patient with a final clinical diagnosis of enteric fever without laboratory confirmation of *S*. Typhi or *S*. Paratyphi infection through blood culture; Laboratory-confirmed case: a patient with *S*. Typhi or *S*. Paratyphi isolated from the blood by culture	Laboratory and clinical records of hospitalized laboratory-confirmed cases
India	Postgraduate Institute of Medical Sciences, Chandigarh; Medanta Hospital, New Delhi; Apollo Hospital, Kolkata; Kasturba Medical College – Manipal University Hospital, Manipal and Christian Medical College, Vellore	January 2014– December 2015	Laboratory-confirmed case: a patient with *S*. Typhi or *S*. Paratyphi isolated from the blood by culture; Surgical case: a patient with intestinal perforation regardless of blood culture confirmation	Laboratory records of laboratory-confirmed cases and clinical records of hospitalized laboratory-confirmed cases and surgical cases
Nepal	Bayalpata Hospital, Achlam; Damauli Hospital, Vyas; Dhulikhel Hospital, Kavrepalanchowk; and Kirnetar Health Center, Dolakha	August 2013– June 2016	Suspected case: a patient >12 months of age presenting to the study sites with a self-reported >72-hour history of fever; Laboratory-confirmed case: a patient with *S*. Typhi or *S*. Paratyphi isolated from blood by culture	Questionnaires administered to participants, clinical records, and laboratory records of suspect and laboratory-confirmed cases
Pakistan	Aga Khan University Hospital and Aga Khan Hospital for Women and Children, Karachi; Aga Khan Hospital Hyderabad, Hyderabad	January 2012– December 2014	Laboratory-confirmed case: a patient with *S*. Typhi or *S*. Paratyphi isolated from the blood by culture	Laboratory and clinical records of hospitalized laboratory-confirmed cases

Abbreviations: *S*. Paratyphi, *Salmonella enterica* subspecies *enterica* serovar Paratyphi; *S*. Typhi, *Salmonella enterica* subspecies *enterica* serovar Typhi.

### Data Collection and Analysis

#### Laboratory Data Collection

For each month of data collection, the following information was obtained from laboratories: the number of blood cultures performed, the number of blood cultures positive for *S.* Typhi and *S.* Paratyphi, and any available patient-level demographic information, such as age, sex, neighborhood of residence, and specimen collection date. For the retrospective phase, patient address was only collected up to the smallest administrative unit, to map the catchment area. For Bangladesh and Nepal, data were extracted retrospectively from prospective studies already in place.

#### Inpatient Department Data Collection

Using hospital record numbers and where available, study staff obtained charts of hospitalized participants with laboratory-confirmed *S*. Typhi or *S*. Paratyphi infection and abstracted clinical information, including demographic characteristics, treatments, investigations, clinical manifestations, and patient outcomes.

Data were abstracted using standard instruments and entered electronically for analysis, which was performed at the country level. Differences in sex and age distributions were analyzed using Wilcoxon rank sum tests and differences in proportions. Blood culture positivity rates and antimicrobial resistance patterns were descriptively reviewed. More details about the analyses performed are available in the country-specific articles published in this supplement [9, 12–14].

### Ethical Review

The study protocols were reviewed and approved by the Centers for Disease Control and Prevention (Atlanta, GA); institutional ethics committees at Translational Health Science and Technology Institute (Faridabad, India), the Postgraduate Institute of Medical Sciences (Chandigargh, India), Medanta Hospital (Gurugram, India), Apollo Gleneagles Hospital (Kolkata, India), Kasturba Medical College – Manipal University Hospital (Manipal, India); the Institutional Review Board at Christian Medical College (Vellore, India); the Institutional Review Board for Human Subjects Research of the Nepal Health Research Council (Kathmandu, Nepal); the Kathmandu University School of Medical Sciences Institutional Review Committee (Dhulikhel, Nepal); Partners Human Research Committee (Boston, MA); the University Health Network Research Ethics Board (Toronto, Canada); the Stanford University Institutional Review Board (Stanford, CA); the Ethical Review Committee of Aga Khan University (Karachi, Pakistan); and the Ethical Review Committee of Bangladesh Institute of Child Health (Dhaka, Bangladesh).

Identifying personal information was accessible only to evaluation staff at the sites. All data reported to investigators were deidentified.

## RESULTS

Overall results from sites in each country, including the number of blood cultures and blood culture positivity rate (total and by organism), are shown in Table 2 and described below. At the Bangladesh hospitals, 51923 children were admitted to the site hospitals, and of those admitted, 15917 (31%) had blood cultures performed. Nearly 3% (443 of 15917) were culture positive for significant bacterial growth, and 63% of these isolates (279 of 443) were confirmed to be *S*. Typhi (241 [86%]) or *S.* Paratyphi (38 [14%]). An additional 1591 children were suspected as having enteric fever, yielding 1870 children with suspected or confirmed enteric fever. This represents 3.6% of all hospital admissions (1870 of 51923) in both hospitals in the 2-year period. At all 5 hospitals in India, 267536 blood cultures were performed during the study period, and of these, 1418 (0.53%) were positive for *S*. Typhi (1147 [81%]) or *S*. Paratyphi (271 [19%]). In the 4 Nepal sites, 4309 participants were enrolled as having suspected infection, of whom 176 (4.1%) had a blood culture positive for pathogenic bacteria; 109 (62%; 2.5% of participants with blood cultures performed) had cultures positive for *S.* Typhi (87 [80%]) or *S.* Paratyphi (22 [20%]). Among the 133017 blood cultures performed in the hospitals in Pakistan, 2872 (2.2%) were positive for *S.* Typhi (1979 [69%]) or *S.* Paratyphi (893 [31%]).

**Table 2.** Blood Culture Positivity Rate Among Individuals With Enteric Fever During Each Country-Specific Study Period, Bangladesh, India, Nepal, and Pakistan, Surveillance for Enteric Fever in Asia Project (SEAP) Phase I, 2012–2016

Country	Duration of Data Collection, Mo	Blood Cultures Performed, No.	Positive Blood Culture Results, No. (%)		
			*S.* Typhi Detected	*S.* Paratyphi Detected	Overall
Bangladesh	24	15 917	241 (1.5)	38 (0.24)	279 (1.75)
India	24	267536	1147 (0.43)	271 (0.1)	1418 (0.53)
Nepal	34	4309^a^	87 (2)	22 (0.5)	109 (2.5)
Pakistan	36	133017	1979 (1.49)	893 (0.67)	2872 (2.2)

^a^Data are for participants enrolled in an ongoing prospective surveillance study, using a case definition of ≥3 days of fever.

**Figure 1.**
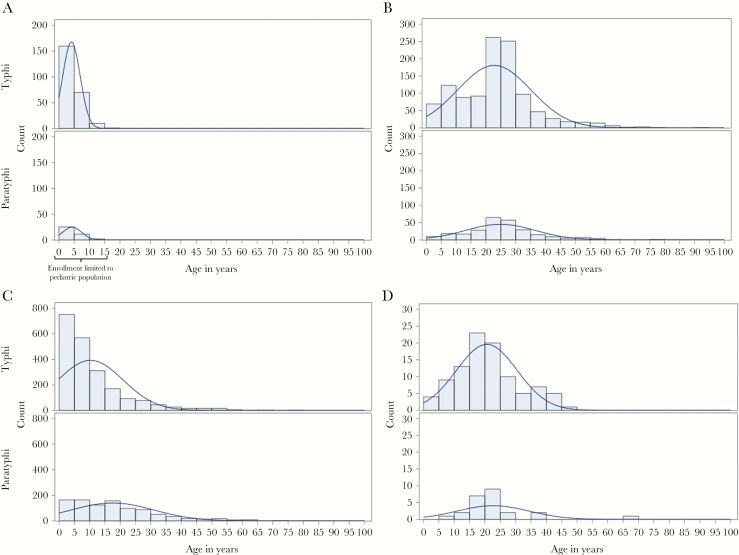
Age distribution of enteric fever cases in Bangladesh (*A*), India (*B*), Pakistan (*C*), and Nepal (*D*), Surveillance for Enteric Fever in Asia Project (SEAP) Phase I, 2012–2016. *S*. Paratyphi, *Salmonella enterica* subspecies *enterica* serovar Paratyphi; *S*. Typhi, *Salmonella enterica* subspecies *enterica* serovar Typhi.

The age distributions of participants with laboratory-confirmed cases varied between the 4 countries (see Figure 1). Bangladesh had the youngest participants for both *S*. Typhi (median, 3 years; interquartile range [IQR], 2–6 years) and *S.* Paratyphi (3 years; IQR, 2–6 years) infection, but this was expected as the 2 site hospitals served pediatric patients only. In Pakistan, which also captured more pediatric cases, participants with *S*. Typhi infection were significantly younger, compared with *S.* Paratyphi–infected participants (median, 7 years [IQR, 3–13 years] vs 15 years [IQR, 6–25 years]; *P* < .0001). Nepal, however, had age distributions curves shifted toward young adults (median, 19 years [IQR, 14–26 years] for *S.* Typhi infection and 20 years [IQR, 17–24 years] for *S.* Paratyphi infection), as did India (median, 24 years [IQR, 14–28 years] for *S.* Typhi infection and 24 years [IQR, 18–30 years] for *S.* Paratyphi infection). The difference in ages between organisms was significant in India (*P* = .01) but not in Nepal. In all 4 countries, the sex of the majority of the participants was male (68% in India, 61% in Pakistan, 55% in Bangladesh, and 52% in Nepal).

**Figure 2.**
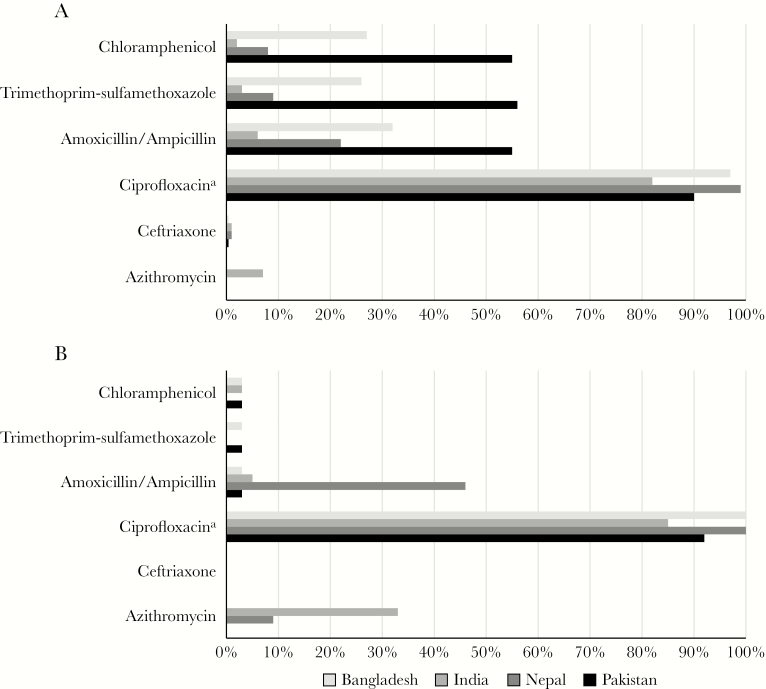
Antimicrobial resistance patterns among *Salmonella enterica* subspecies *enterica* serovars Typhi (*A*) and Paratyphi (*B*) isolates in Bangladesh, India, Nepal, and Pakistan, Surveillance for Enteric Fever in Asia Project (SEAP) Phase I, 2012–2016. ^a^Reduced susceptibility; nalidixic acid was used as a proxy in Bangladesh.

Antimicrobial resistance patterns for each country, by organism, are shown in Figure 2. Overall, a higher proportion of *S.* Typhi isolates were resistant to chloramphenicol, ampicillin, and trimethoprim-sulfamethoxazole, compared with *S.* Paratyphi isolates. A high proportion of both *S.* Typhi and *S.* Paratyphi isolates from all 4 countries were nonsusceptible to ciprofloxacin, and a number of isolates exhibited resistance to ceftriaxone (1 of 115 *S.* Typhi isolates in Nepal and 4 of 418 in India) and azithromycin (2 of 23 *S*. Paratyphi isolates in Nepal and 2 of 30 *S.* Typhi isolates and 1 of 3 *S*. Paratyphi isolates in India).

Complete results are available in the country-specific articles published in this supplement [9, 12–14].

## DISCUSSION

This study generated data on enteric fever prevalence among febrile patients, by age, sex, and time (month/year), along with information on other clinical factors and antimicrobial resistance among hospitalized participants with enteric fever from sentinel sites in 4 countries, allowing for cross-site comparison. These results were critical to informing data collection for the prospective phase of the SEAP. While prospective surveillance will occur in India through a separate surveillance system (the National Surveillance System for Enteric Fever in India), we have identified sites in Bangladesh, Nepal, and Pakistan that continue to experience a sustained burden of enteric fever and are capable of contributing useful information on disease burden in prospective surveillance.

In Pakistan, *S*. Typhi infections were concentrated around younger ages, and while Bangladesh had pediatric cases only, because the sites were pediatric hospitals, nearly a quarter of *S*. Typhi–infected participants were <2 years old. This is an important observation, since these ages represent a population that can be protected with TCVs if a vaccination program is implemented. While overall the burden has shifted to an older population in India and Nepal as compared to Pakistan and Bangladesh, there remains a considerable burden of *S.* Typhi at the younger ages and a need for TCV implementation; the older population would also benefit from the WHO’s recommendation for a catch-up campaign beginning at 15 years [3]. These differences in ages should be viewed in the context of the study design, though, because the differences could be partially explained by trends in healthcare-seeking behavior. This is also the case for sex distribution; literature has shown that generally a higher proportion of males seek care at the hospital, especially in younger ages [15]. Prospective surveillance performed in these countries will allow us to adjust incidence estimates based on reported care-seeking behavior and to estimate rates of infection by sex and age group.

We were also able to review current patterns of antimicrobial resistance in all 4 countries. There is still a high proportion of *S.* Typhi isolates resistant to chloramphenicol, ampicillin, and trimethoprim-sulfamethoxazole, especially in Pakistan and Bangladesh [12, 14]. Since the proportion of isolates in these countries that are nonsusceptible to ciprofloxacin is nearing 100%, this leaves third-generation cephalosporins, azithromycin, and carbapenems as the last remaining oral antibiotics for treating typhoid. As we have identified a few isolates in Nepal and India resistant to these agents [9, 13], continued close monitoring of antimicrobial resistance is essential to inform appropriate treatment recommendations.

SEAP Phase I has yielded valuable information on the enteric fever burden across multiple settings in South Asia, but the results are best considered in the context of the chosen study settings. There may be an upward bias in incidence estimates generated in urban populations when compared with rural populations, likely because of transmission drivers such as population density and scarce access to clean water and sanitation [16]. Indeed, data from the Nepal study described in this supplement found that the prevalence of enteric fever among febrile individuals was strongly correlated with population density [9].

In addition to limitations in in-country generalizability due to the selected sites, SEAP Phase I provides an important perspective on key issues and limitations of retrospective data. Data obtained were limited in terms of comparability across settings and period. The data from each setting were highly variable with regard to availability, including variability for the surveillance period, age groups included in surveillance, and surveillance methods. Perhaps most significant was the variability in case definitions used for participants with suspected cases for whom a blood culture was requested. The lack of standardized criteria for requesting blood culture makes the diagnosis of enteric fever highly subjective to the treating clinicians’ opinions. This is illustrated in Nepal, where half of individuals with a fever duration of >3 days were given a provisional clinical diagnosis of typhoid, but fewer than 5% of these individuals had culture-confirmed infection [9]. Furthermore, clinicians in resource-poor settings may be less likely to prescribe testing for poorer patients, who are possibly at a higher risk of enteric fever and subsequent poor outcomes. Additionally, hospital-based studies have an inherent limitation in that they underestimate the disease burden in the community, owing to differential health-seeking behaviors [17]. Active, community-based surveillance, is also more likely to be able to identify complications and sequelae that occur in patients with typhoid fever after discharge from a hospital or treatment at an outpatient clinic. These limitations and others [18], combined with nonstandardized case definitions, have important implications for disease burden and incidence estimations, which form the basis of national and global policies [1, 2]. The data from Phase I were also limited to what was available retrospectively through existing data sources, primarily clinical records from a few sites. While laboratory data were relatively more complete and available, linking laboratory and clinical data sources presented difficulties. Clinical records were either missing or did not adequately capture the required information for a large proportion of laboratory-confirmed cases, specifically among individuals seen in outpatient departments. This prohibited sufficient characterization of the clinical profile of laboratory-confirmed cases and differences in clinical profiles by antimicrobial resistance. This is an especially critical lesson learned in the era of rising antimicrobial resistance and a potentially increased severity of illness associated with resistant strains [19–21].

Phase II of the SEAP will address many of these limitations inherent to retrospective study design. For example, the protocol for Phase II has a clearly defined study population, using a created catchment area to define eligibility, and standardized case definitions, which differ by enrollment location within the hospital (eg, outpatient, inpatient, and surgical settings). We also plan to prospectively collect robust data through multiple sources, including patient interviews, laboratory records, and medical charts, to improve the quality and amount of available information and its potential to be appropriately linked and analyzed. Unlike Phase I, Phase II will be able to estimate incidence on the basis of data from hospital-based surveillance, using the low-cost hybrid method outlined by Luby et al [17]. We plan to obtain the proportion of participants with disease matching our case definition who sought healthcare and create age-stratified incidence by using healthcare-seeking behavior, as well as to adjust for a variety of factors related to potential biases as described by another article in this supplement [22], to deliver more-discrete and accurate estimates to country governments and decision makers.

SEAP Phase I is one of the few multicenter studies in South Asia that have attempted to retrospectively assess the enteric fever burden by using existing clinical and disease surveillance systems. With investment in capacity building, SEAP is building a low-cost and sustainable surveillance system that can be leveraged for ongoing surveillance to evaluate the impact of interventions, including vaccines, by integrating data capture into existing site infrastructure. It is unlikely that vaccines will entirely eliminate typhoid, shown by modeling in South Asia, but that a combination of water, sanitation, and hand hygiene interventions and vaccination may eliminate typhoid in settings where it is endemic [23]. Since the recent availability of a licensed and WHO-prequalified typhoid conjugate vaccine (Typbar-TCV; Bharat Biotech, India), which has been shown to be immunogenic in children aged <2 years, demonstrating vaccine effectiveness and public health impact is more important than ever [5, 24–26]. SEAP Phase II will produce not only accurate estimates of the burden of disease in selected areas in Bangladesh, Nepal, and Pakistan, but also baseline data for future vaccine effectiveness studies in the site catchment areas, as well as evaluate the impact of additional long-term interventions, such as improvements in water and sanitation infrastructure.

## Notes

***Acknowledgment***. This article is part of the supplement “Surveillance for Enteric Fever in Asia Project,” sponsored by the Sabin Vaccine Institute.

***Financial support***. This work was supported by the Bill and Melinda Gates Foundation (grant OPP11130007).

***Supplement sponsorship.*** This article is part of the supplement “Surveillance for Enteric Fever in Asia Project,” sponsored by the Sabin Vaccine Institute.

***Potential conflicts of interest***. All authors: No reported conflicts. All authors have submitted the ICMJE Form for Disclosure of Potential Conflicts of Interest. Conflicts that the editors consider relevant to the content of the manuscript have been disclosed.
