# Erratum

**DOI:** 10.1093/infdis/jiy613

**Published:** 2018-10-29

In “Phase I of the Surveillance for Enteric Fever in Asia Project (SEAP): An Overview and Lessons Learned” [J Infect Dis 2018; https://doi.org/10.1093/infdis/jiy522], the heading “Study Design and Objectives” has been removed. In Table 1, the text “Aga Khan University Hospital and Kharadhar General Hospital, Karachi” should be “Aga Khan University Hospital and Aga Khan Hospital for Women and Children, Karachi; Aga Khan Hospital Hyderabad, Hyderabad”. The Acknowledgment text has been moved under the heading “Supplement sponsorship”.

