# Erratum

**DOI:** 10.1093/infdis/jiy694

**Published:** 2018-12-10

Corrigendum to “Phase I of the Surveillance for Enteric Fever in Asia Project (SEAP): An Overview and Lessons Learned” by Barkume et al. *The Journal of Infectious Diseases*, jiy522, https://doi.org/10.1093/infdis/jiy522.

The text “Nepal, however, had age distributions curves shifted toward young adults (median, 14 years [IQR, 19–26 years] for S.Typhi infection and 17 years [IQR, 20–24 years] for S.Paratyphi infection), as did India (median, 14 years [IQR, 24–28 years] for S.Typhi infection and 18 years [IQR, 24–30 years] for S.Paratyphi infection).” has been replaced with the text “Nepal, however, had age distributions curves shifted toward young adults (median, 19 years [IQR, 14–26 years] for S.Typhi infection and 20 years [IQR, 17–24 years] for S.Paratyphi infection), as did India (median, 24 years [IQR, 14–28 years] for S.Typhi infection and 24 years [IQR, 18–30 years] for S.Paratyphi infection).”

